# Gut Microbiota Modulators Based on Polyphenols Extracted from Winery By-Products and Their Applications in the Nutraceutical Industry

**DOI:** 10.3390/life14030414

**Published:** 2024-03-20

**Authors:** Laura-Dorina Dinu, Emanuel Vamanu

**Affiliations:** Faculty of Biotechnologies, University of Agricultural Sciences and Veterinary Medicine, 011464 Bucharest, Romania; laura.dinu@biotehnologii.usamv.ro

**Keywords:** viticulture and vinification by-products, grapevine polyphenols, gut microbiota modulation, dysbiosis, nutraceuticals

## Abstract

Vine-growing for the production of wine is one of the oldest and most important agricultural activities worldwide, but the winemaking process leads to vast amounts of waste. Viticulture and vinification by-products have many bioactive molecules, including polyphenols, prebiotic fibers, organic acids, and minerals. While research on the specific human health effects of grapevine residues (pomace, seeds, barks, stalks, canes, and leaves) is still ongoing, the available data suggest the potential to positively modulate the normal and dysbiotic gut microbiota (GM) using polyphenol-rich extracts obtained from winery by-products. This review provides an updated summary of the in vitro and in vivo evidence in animal models and humans concerning the ability of polyphenol-rich winery residue to be used as a GM modulator that supports their nutraceutical applications as a functional ingredient. Additionally, this review aims to enhance interest in viticulture waste (grapevine stems and leaves), as the levels of polyphenols are similar to those found in red grapes or seeds. However, more research is still needed to obtain innovative products. The valorization of winery residues is not only environmentally friendly; it can also be economically beneficial, creating added-value nutraceuticals that modulate microbiota and a new revenue stream for wine producers.

## 1. Introduction

In recent years, the intersection of human microbiota research and sustainable waste management practices has opened new directions for addressing environmental challenges and the health industry, where obtaining bioactive compounds from agro-waste is a hot topic. For the wine industry, the abundance of bioactive molecules in viticulture and winery by-products (grapevine stems and leaves, grape seeds and skins, wine pomace, and bagasse), especially dietary fibers and polyphenols, make these a vital source for obtaining add-value products for health. It is estimated that waste generated during grape juice and wine production constitutes up to 30% (*w*/*w*) of fresh grape weight and is related to grape variety, viticulture practices, and the winemaking process [1]. All vine and grape-derived subproducts contain polyphenols that plants produce as secondary metabolites to defend against environmental and pathogen aggression [2]. The potential health impact of polyphenol-rich viticulture and winery by-products has become an increasingly investigated research topic, primarily regarding their ability to modulate gut microbiota and, thus, generate beneficial effects in human and animal hosts. Also, consumers’ growing awareness regarding healthy lifestyles and their concerns about the protection of the environment have helped expand the research about transforming agro-waste into healthy products. At the same time, the rising popularity of grape pomace (GP) among manufacturers as a cheap and healthy ingredient has expanded the market for grape pomace and seeds, and new GP-fortified foods and dietary supplements have been released [3].

The human gut microbiota (GM) or gut microflora is composed of bacteria, fungi, archaea, and viruses, and it has up to 1000 different microbial species [4,5]. Bacteria are the largest and best-studied component and include two major phyla, Firmicutes and Bacteroidetes (constituting over 90% of all bacterial species), followed by the phyla Actinobacteria at 3% and Verrucomicrobia at 2% [4,5]. In the human body, dietary polyphenols have low bioavailability and, during their gastrointestinal transit, interact with digestive enzymes and gut microbiota [4,6,7]. The current literature consistently indicates that ingested polyphenols have a prebiotic-like effect and positively modulate the composition and functionality of gut microbiota while changing intestine homeostasis. Moreover, the relationship between polyphenols and microbiota is bidirectional, as the GM can metabolize phenolic compounds into low-molecular-weight molecules with higher bioavailability [6,8]. Considering the large proportion of unabsorbed diet polyphenols, it is becoming evident that the favorable health effects from the intake of polyphenols are more closely related to the GM composition and the production of microbial-derived metabolites with higher bioavailability than to direct absorption by the human cells [9].

Current research has proved the health benefits of extracts and foodstuffs obtained from winery wastes, including antioxidant, anti-inflammatory, cardioprotective, antidiabetic, and anticancer properties, and so on [10]. However, only a few works have focused on the effect of grapevine residues on GM composition and the microbial metabolic profile, and even fewer show the correlation between GM changes and the health effects reported. Recently, works uncovered the modulator effects of grape pomace, considered the main agro-waste used in the formulation of functional additives in feedingstuffs, dietary supplements, and fortified foods [3,10,11]. Therefore, this study aimed to review and update the research on microbiota modulation using polyphenol-rich extracts and diets supplemented with winery by-products (grape pomace and seeds). In addition, this study documents whether viticulture waste (grape canes and leaves) can be a valuable and suitable source of polyphenols, enhancing the interest in its use in obtaining high-value functional ingredients. The synergy between waste valorization and nutraceutical product development exemplifies a holistic approach to addressing environmental and health challenges. Thus, this review strongly advocates valorizing winemaking secondary products in different nutraceutical applications.

Our methodology was used to search the available online databases (Web of Sciences, Scopus, Springer Link, Wiley, ScienceDirect, and Google Scholar) for studies involving grapevine waste (polyphenol composition, isolation methods, and applications), diets supplemented with winery by-products, and gut microbiota–dietary polyphenol interactions. The primary search keywords were grapevine waste (grape pomace, marc, seeds, skins, stalks, bagasse, vine stems, canes, shoots, trunk, roots, and leaves); grape and vine polyphenols; and gut microbiota, health benefits, food, and nutraceutical applications. Succeeding the primary search, the results were evaluated using a hierarchical approach based on the title, abstract, and full manuscript and excluding letters to editors, editorials, book reviews, and protocols. In vitro research focused solely on the effect of grapevine extracts on specific microbial strains was excluded. All selected and eligible articles were evaluated considering their content, year of publication, and relevance for the present review.

## 2. Effects of Polyphenol-Rich Winemaking By-Products on Gut Microbiota Composition and Function

In the human model, dietary polyphenols have limited absorption in the stomach and the small intestine. In contrast, most polyphenols during intestinal transit interact with enzymes and the GM and are eventually excreted in feces. High-molecular-weight polyphenols, found in tremendous amounts in winemaking waste, such as proanthocyanidins, allocated catechins, and anthocyanidins, have lower bioavailability mainly due to glycosidic linkages that limit their absorption [12]. It is essential to mention that, during their gastrointestinal transit, polyphenols from food or dietary supplements are transformed into structurally different phenolic forms compared with intake compounds [8]. Many studies indicate that unabsorbed polyphenols (90–95% of polyphenol intake) reach the large intestine and interfere with the GM, which modifies the microflora community’s composition and functioning, generates various microbial metabolites, and influences intestinal homeostasis [3,4,7,11]. In the colon, polyphenolic compounds enhance the abundance of specific bacterial groups acting as prebiotics while inhibiting others based on their antimicrobial potential. Some studies have reported that grape-based waste polyphenols increase beneficial taxa, such as *Lactobacillus* spp., *Akkermansia* spp. and *Bifidobacterium* spp., which improve gut barrier protection, or *Faecalibacterium prausnitzii* and *Roseburia* spp., which are associated with an anti-inflammatory effect [13,14,15,16,17]. At the same time, pathobionts (e.g., *Escherichia coli*, *Enterobacter cloacae*, and *Clostridium*) and food pathogen species are dominant [11,17,18]. At the molecular level, food polyphenols have been reported to interfere with bacterial cell-to-cell communication (quorum sensing) and affect the cell membrane permeability of pathogens, which are important features that help fight against pathogen infection [19]. In the gastrointestinal tract, polyphenol glycosides are cleaved and metabolized by the intestinal microbiome to produce low-molecular-weight and more highly absorbable bioactive metabolites than their precursors. Polyphenols and their more bioavailable and biologically active metabolites enter the host’s blood circulation and appear to be responsible for reported pleiotropic health effects. Thus, the microbial symbionts increase the energy and nutrient extraction from diet and influence the host’s signaling pathways [7]. The primary phenolic microbial-derived metabolites from winery waste polyphenols are benzoic, phenylpropionic, phenylacetic, and hippuric acids [11,14]. However, the number of bacterial strains isolated on culture media and capable of using polyphenols as natural substrates is low [19]. Primary microbes from the GM that metabolize GP phenolics, such as strains of *Bacteroides* spp., *Clostridium* spp. and Enterobacteriaceae, are both commensal strains and opportunistic pathogens [14]. At the same time, the dietary fraction of vine grape waste is fermented by the GM. It generates microbial metabolites, such as short-chain fatty acids (SCFAs), which can influence intestinal ecology and human health after being released into the blood circulation. The relationship between polyphenols and the GM is influenced by the GM composition, type, and quantity of polyphenol molecules involved in this interaction. Therefore, long-term treatments seem beneficial [10,11,14]. A remarkable and consistent amount of experimental data have powerfully demonstrated the role of the GM as a critical component in understanding the beneficial health effects of dietary polyphenols. However, the precise mechanisms involved deserve further clarification.

### 2.1. Grape Pomace Extract

The main subproduct of the winemaking industry is grape pomace or grape marc (the French word for pomace), which is generated in large quantities during the vinification process after pressing grapes to produce must and wine. It is estimated that, worldwide, the wine industry produces approximately 8.49 million tons/year of GP, which is mainly used as a supplement in animal feed or transformed into ethanol by distilleries, according to European Council Regulation EC 479/2008 [20,21]. Grape pomace and lees have phytotoxic effects if applied to crops or land. Thus, proper waste management measures are important for avoiding environmental contamination [22]. Therefore, new applications have been developed in the pharma, cosmetics, and fortified food industries. This solid subproduct of vinification is composed of husks, seeds, pulp, stalks, and leaves, and it is characterized by its high dietary fiber and polyphenolic compound composition [20]. While pomace represents only 20–30% of the grape weight, more than 60–75% of polyphenols are found in grape pomace, seeds, and skins [23]. It is important to mention that the total polyphenol content (TCP) mainly refers to the free phenolics solubilized in the organic solvent used for extraction, but some phenolics are bound to polysaccharides. Various studies have found the TCP of grape pomace to be approximately 60–75 mg GAE/g, while the total anthocyanin content can be up to 131.4 mg/100 g [2,24,25,26]. The variation in the concentration of GP polyphenolic compounds is correlated with the grape variety; the cultivation conditions and harvesting time; and the parameters of the winemaking processes [2]. The extraction parameters, especially the solvent type, temperature, and extraction time, must be optimized to maximize the recovery of the natural phenolic compounds from GP [27,28]. The main phenolic components of grape marc are anthocyanins (only in red grape pomace), flavonols, stilbenes, and phenolic acids [10,20]. Grape skins or husks are the significant components of GP, with 28 and 56% of the dry matter in white and red pomace, respectively [29]. Anthocyanins are mainly found in the inner layer of grape husks, along with tannins and small quantities of gallates [30]. Grape seeds (GSs) constitute up to 50% of the grape marc dry matter and contain approx. 7% *w*/*w* polyphenols, while grape stalks constitute around 6% *w*/*w* polyphenolic compounds, mainly tannins associated with lignin, flavan-3-ols, flavonols, gallic and syringic acids, and stilbenes [31,32]. For human health, phenolics are the most valuable bioactive molecules from GP, which are in higher amounts in red pomace than white ones, followed by dietary fibers. At the same time, both can serve as prebiotics [2,3,20].

The abundance of natural phytochemicals with health benefits, the low cost of grape marc processing, and the implementation of the circular economy strategy have increased interest in studying the effects of GP polyphenols on the regular and dysbiotic microbiota. In the last decade, some in vitro and in vivo studies of animals (pigs, mice, rats, calves, and lambs) and humans have proved the GM modulation effect of grape pomace extracts and supplemented diets (Table 1). Compared with recently published reviews concerning the impact of GP and GS on GM modulation, we found a higher number of works. We targeted the data that support their valorization in nutraceutical production. Two of these studies compared GP and GS extracts using the same animal-based model, while eighteen were published between 2020 and 2023, proving the increased interest in this subject. [13,15]. However, we noticed that most of the research and clinical trials have focused on the interaction between polyphenols from grapes, non-fermented derivates (grape juice), and wine with gut microbiota. Exhaustive reviews on this subject were recently published. Still, many mysteries remain regarding the bioavailability of these polyphenolic compounds, but many mysteries remain regarding the bioavailability of these polyphenolic compounds and their claimed health effects [33,34,35].

Human digestion is a dynamic and complex multistage process; therefore, in vitro, simulated digestion methods are widely used systems that offer an alternative way to simulate digestibility and study modulator effects on the GM in animals and human models. Their main advantages are their low cost, reproducibility, and efficiency. Small animal studies that use rodents that have ingested different quantities of GP supplemented in their diets are important precursors, providing information for human trials [3]. To prove that GP is a valuable feed additive, studies have studied its effect on animal welfare. All these studies have pointed out the potential to use GP as a GM modulator. Still, there are numerous differences between the reported in vivo results that could be attributed to variations in the composition of GP-derived products, doses, targeted populations, inter-individual variability, etc. Using gastrointestinal simulators, different in vitro studies prove that the relationship between the gut microbiota and unabsorbed phenolic compounds from GP is complex.

An important feature of a functional ingredient is its stability during gastrointestinal transit so it can reach the large intestine in adequate amounts to modulate the GM. GP studies on the stability and bioavailability of GP phytochemicals during gastrointestinal transit have shown that the number of intake phenolics decreases. In contrast, new and more minor phenolics induced by degradation have been detected in the intestine [48,49,50]. Wang et al., 2017, noticed that phenolic compounds were stable under gastric conditions, but pancreatic digestion significantly decreased TPC and antioxidant activity, while flavonols and anthocyanins were more sensitive [51]. However, other in vitro research has found that large phenolic compounds reach the colon and impact the GM [14]. Alginate encapsulation and food matrix delivery have been used to optimize and improve phenolic stability during gastrointestinal transit [52].

The prebiotic effect on beneficial GM bacteria is another important feature of any microbiota modulator. It is important to mention that, in the case of grape marc, phenolic compounds, and oligosaccharides promote bacterial growth, but no studies have analyzed this effect separately. In vitro works have noticed the positive effects of GP on beneficial bacterial taxa from GM (e.g., *Lactobacillus* spp. and *Bifidobacterium* spp.), which has also been observed by some studies of livestock animals and rats [14,15,17,18,43,46]. Chacar et al., 2018, reported that GP promoted a 21–27% increase in *Bifidobacterium* spp. at low doses of GP (up to 5 mg/kg body weight/day); at higher doses, this beneficial strain stopped the growth and reached a plateau, while *Lactobacillus* spp. decreased [16]. The growth of bifidobacteria was correlated with the presence of two microbial metabolites in urine samples among 16 detected metabolites [17]. However, other research with humans and rats has concluded that lactic bacteria decrease [38,47]. GP modulates the GM mostly at the genus level, increasing biodiversity in long-term treatments [15,16,45]. *Bacteroides* spp. have an important role in maintaining gut homeostasis and intestinal barrier integrity and are known to use phenolic compounds from GP; therefore, many studies have noticed its increase, as well as for other taxa related to the degradation of GP constituents (*Eubacterium* spp. and *Ruminiclostridium* spp.) [14,15,45,46]. Similarly, some commensal taxa from Firmicute phyla, e.g., *Enterococcus* and *Roseburia*, have been reported to enlarge in vitro and in animal studies [15,36,47]. The administration of GP and GS extracts after antibiotic treatment in mice showed a higher relative abundance of bacteria belonging to the Verrucomicrobiota and *Akkermansia* spp., which are linked to improved barrier function [13].

The antimicrobial effect on opportunistic pathogens from the GM or different pathogenic species has been found in different in vivo studies. GP-supplemented diets inhibited pathogen populations of *E. coli* and other Enterobacteriaceae in lamb and piglets in [18,42]. *Campylobacter jejuni* growth decreased in piglets but not lamb, while *Streptococcus* spp. and *Treponema* increased in pigs fed GP in [18,41,42]. Incorporating GP concentrate in broiled chickens’ diets increases *E. coli* and *Clostridium* in the cecum, though the latter also decreases in the ileum [15]. However, in a study of pigs infected with *Ascaris suum*, diet supplementation with GP did not decrease the number of parasite larvae, while in vitro results have shown the antihelmintic effect of GP [40]. Still, a bioactive diet can modulate the GM’s composition. It can change its colonic branched-chain SCFA concentration while also significantly improving the immune response in infected pigs, increasing the number of eosinophils and mast cells in pig intestine mucosal [40]. In small animals, *Clostridium perfringens* (Cluster I) inhibition after long-term GP administration was correlated with microbial metabolite Daidzedin, which was detected in urine samples only after 14 months post-treatment in [16,17]. Similarly, Taladrid et al., 2022, reported a reduction in *Clostridium* I and *Streptococcus* populations in patients with high cardiometabolic risk after GP seasoning consumption [11]. An increase in antimicrobial peptides was detected after GP consumption in mice fed a high-fat diet for eight weeks in [47].

The ability of GP polyphenols to modulate gut microbiota composition and dynamics and, subsequently, the metabolic profile has been found by different in vitro and in vivo studies. In the case of grape marc, the phenolic compound forms are esters, glycosides, and polymers, which suffer various degradations caused by intestinal enzymes and/or gut microbiota before absorption [14]. Two in vitro studies on the digestion of GP extracts and bioavailability detected 16 and 21 phenolic metabolites, while the main absorbables are different benzoic, phenylacetic, and phenylpropionic acids [14,36]. Research about the fate of ingested polyphenols from GP is rare, and only one study with rats linked the presence of microbial metabolites produced following the long-term consumption of GP to their effect on the GM [17]. The detection of two phenolic acid derivates, 3-hydroxyphenyl acetic acid and 2-(4-hydroxyphenyl) propionic acid, was correlated with *Bifidobacterium* growth after the consumption of GP (up to 5 mg/kg body weight/day), while the phenolic sulfate derivative of Daidzedin with *C. perfringens* caused inhibition [17].

Interestingly, at higher GP concentrations, these metabolites were not detected in urine samples from rats, and bacterial growth stopped. Also, the authors suggested that a synergistic action between different metabolic products might be responsible for the observed effects. Changes in short-chain fatty acid composition as a result of fermented dietary fiber were reported by most of the studies [14,37,40]. GP-derived nutraceutical products were reported to reduce trimethylamine-N-oxide (TMAO), a gut microbiota-derived metabolite strongly related to cardiovascular disease [53]. However, the potential modulatory effects of the product on GM were not analyzed in this case.

Small animal studies and human trials have shown that GP has the potential to modulate normal microbiota during a pathological situation (e.g., antibiotic treatment or obesity), especially after long-term GP administration. Moreover, GP administration helps the recovery of the GM after antibiotic treatment in obese mice and improves gut barrier function [13,47]. In this case, a GP-supplemented diet partially correlates to GM modification, which promotes a reduction in fat mass gain and adipose tissue inflammation, including beneficial effects on glucose homeostasis. There is a growing interest in evaluating the anti-hyperglycemic effect of GP, as studies on mice and different human experimental trials have suggested [14,37,47,54]. Recently, a human trial with high-cardiovascular-risk subjects noted that GM modulation might mediate the cardiometabolic effects of GP seasoning treatment, which reduced glycemia and decreased blood tension [11]. Despite encouraging outcomes from animal studies, some results from clinical trials are still controversial. The human response to polyphenol supplementation does not seem homogeneous, and inter-individual variability in clinical trials should be considered [37,39]. Thus, a recent study investigated the role of gut microbiota and microRNAs (miRNAs) in homogenous responses to GP polyphenol intake for six weeks using subjects with high cardiometabolic risk and variability in insulin response. The responder subjects proved to have an increased miR-222 level and lower populations of Firmicutes and *Prevotella* spp. [39].

Animal studies with a GP-supplemented diet have highlighted increased health and performance and improved meat quality in livestock animals [41,43,44]. All the studies underline the GM modulatory potential of GP-derived products, both in the composition and functioning of GM, and some correlation with other biological activities (e.g., antioxidant, anti-inflammatory, anti-hyperglycemic, and cardioprotective activities) have been pointed out. However, it takes work to draw definite conclusions about the complex interaction between polyphenol-rich GP-derived products and the GM. Still, more studies are required to understand the mechanisms involved, including the synergistic effects of different polyphenol classes.

### 2.2. Grape Seed Extract, Oil and Flour

Another valuable subproduct discharged in large quantities (over three megatons/year worldwide) is grape seed, which is obtained from the juice and winemaking processes [55]. The number and weight of seeds are variable depending on the grape berry size, and the seeds represent about 5% of the grape’s weight [56]. The exhausted grape marc (without its alcoholic and tartaric constituents) is the starting point of GS-derived products. First, GP is screened and sieved to obtain GS, which is dried and pressed to extract oil, while the defatted GS produces flour [55,56]. The GS waste composition is complex, with some valuable components, mainly various polyphenolic compounds (5–8%), vegetable oil, natural fibers, proteins, carbohydrates, and minerals [55,56]. Multiple factors, such as grape varieties, climate, soil, or the degree of ripeness, influence the GS waste composition. The pericarp of the seeds is rich in flavonoids. Therefore, seeds are used to prepare grape seed extracts and flour with high amounts of antioxidant proanthocyanidins [55]. Grape seed oil is known for its high essential fatty acids and potent natural antioxidants. Still, only a minor part of the seed’s high phenolic content is transferred to the pressed oil [57,58].

The first study reporting the effect of GS on human fecal flora was published in 2001. Since then, reports have gradually increased. Thus, many in vitro and in vivo studies, mainly on animals, have proven the impact of polyphenol-rich GS extracts on intestinal the microbiota composition and metabolic profiles of healthy subjects and dysbiotics (Table 2). Remarkably, in the last three years, eight papers on the beneficial effects of GS-derived products were published, proving that these subjects are receiving increasing interest from nutritionists and other scientists.

These results follow the ones reported above for GP, though some differences were noted. Comparing the effects of GP and GS extracts on broiled chickens, Viveros et al., 2011, recorded that GP and GS extracts changed the GM from cecum to ileum with slight differences between the effects, while GS extract was reported to decrease weight [15]. Lu et al., 2019, researched the contribution to the recovery of the GM after antibiotic treatment in mice fed a high-fat diet and found that both seeds and pomace extracts restored microbiota. Still, GS had no effect on the beneficial taxa (*Bifidobacterium*, *Lactococcus*, and *Lactobacillus*) [13]. Remarkably, GS can partially restore dysbiosis produced by obesity and inflammatory bowel disease. Studies have shown a correlation between microbiota modulation and beneficial health effects [74,76]. Moreover, many studies of animal models have highlighted an increase in biodiversity indices after a GS-supplemented diet [15,65,68,72].

GS-derived products have proven to be a valuable feed additive for livestock animals, increasing animal growth and improving their health. These effects are associated with GM modulation, especially the prebiotic-like impact on other beneficial taxa and the decrease in detrimental bacteria. For example, based on Spearman’s correlation analysis, Nan et al., 2022, demonstrated a strong correlation between broiler growth performance, abdominal fat percentage, SCFAs, and GM modulation [68]. Moreover, in vitro, rumen fermentation experiments have demonstrated that proanthocyanidin–GS extract could modulate rumen archaeal microbiota, and their administration in diets should be considered to obtain an inhibitory effect on methane emissions [77].

Most small animal studies have used a rodent model of obesity and tried to unveil the link between GS extract benefits and GM alteration in the obesogenic context. A recent study demonstrated that seasonal rhythms might influence GM and GS extract bioavailability [71]. Obese rats were exposed to different photoperiods, but only after 18 h of light GS extract did body weight gain and fat deposits decrease, while GM was strongly altered. The results suggested that seasonal rhythms must be considered for investigations about polyphenol-mediated effects on GM [71]. In all studies, GS administration partially reversed obesity-associated dysbiosis, which increased diversity and beneficial bacteria, normalized the Firmicutes/Bacteroidetes ratio, and reduced pathobiont growth [69,70,72,74]. These GM alterations are linked to decreased body weight and fat accumulation and improved serum lipid parameters, including enterohormone secretions [69,75].

Moreover, Mokrani et al., 2022, showed that grape extracts from seeds and skins could be applied to medication used to treat obesity (orlistat) to increase the beneficial effects [74]. The authors observed that grape extract significantly increases the beneficial microbes *Methanobrevibacter, Ruminococcus* 2, and *Lachnospiraceae* NK4A136, the last two strains being potential probiotics. At the same time, it decreases pathobionts—especially *Streptococcus alactolyticus*/*gallolyticus*, Enterobacteriaceae (*Escherichia* genus), *Allobaculum*, *Turicibacter*, and *Tyzzerella* 3—associated with inflammation, leaky gut, and cardiovascular risk. Unlike with complex grape polyphenols, a GM modulation effect was identified in obese and non-obese rats, and the treatment with orlistat extensively increased *Lactobacillus* spp. but did not improve GM diversity in fat-diet-fed rodents [74]. The work of Zheng et al., 2023, on the canine model suggested that the anti-inflammatory effect of proanthocyanidin-rich GS extract is related to GM modulation and microbiota-derived bile acids [76]. Fecal microbiota was transferred to dogs with mild inflammatory bowel disease to validate that GS extract has a beneficial ingredient with the potential to improve intestinal inflammation [76]. The anti-aging effect of GS extracts is well known in cosmetics. Still, Sheng et al., 2022, proved that a high extract level has more significant potential to delay the aging process via the gut microbiota–liver axis and the gut–microbiota–brain axis [70].

Although there has been a greater number of clinical trials carried out with GS, none provide information about the GM modulation effect. Still, various beneficial effects have been proven that could be linked to gut microbiota activity (e.g., antioxidant, anti-inflammatory, hypolipidemic, and cardioprotective effects). Moreover, there is no work on the effect of GS oil on GM.

## 3. The Potential of Viticulture By-Products to Modulate Gut Microbiota Composition and Function

The major subproducts of vineyards are grape stems (including canes or shoots and stalks), which are usually composted in the field or burned, as they are rich in cellulose, hemicelluloses, lignin, polyphenols, nitrogen, and potassium [78]. It is believed that, annually, 2 to 5 tons/hectare of grapevine canes is produced. However, only in recent years has their valorization received considerable attention, as they are rich in stilbenes and proanthocyanidins [79]. Using a high-resolution mass spectrometry method to analyze the phenolic profile of grape cane extract from the Pinot Noir variety, Escobar-Avello et al., 2019, identified a total of 75 compounds. They revealed the presence of 17 polyphenols not previously described in cane extract [80]. In that study, the most abundant polyphenols were stilbenoids, oligomers, and proanthocyanidins. However, the authors later concluded that the phenolic profile of grape canes obtained using pilot plant extraction differed substantially from those previously obtained in laboratory-scale studies [81]. However, the most abundant phenolic compound class was stilbenoids, mainly E-ε-viniferin, among the 44 phenolics identified. The concentration of stilbene grapevine canes is significantly influenced by vine varieties, the vegetation phase, and growing conditions, including ultraviolet radiation and fungal attacks, as plants produce these secondary metabolites as a response to abiotic and biotic stress [82]. It was reported that, in the eco-dormancy phase, the level of both flavonols (mostly quercetin, quercetin-3-glucuronide, quercetin-3-glucoside, and quercetin-3-galactoside) and stilbenes (trans-resveratrol and ε-viniferin) improves compared with the endo-dormancy phase when flavonols increase, whereas stilbenes decrease [83].

Interestingly, a significant 5-fold increase in stilbene level, especially for t-resveratrol, was reported during the storage of canes for three months after pruning [84]. Other works have noted that six weeks of storage increases stilbene concentrations, while periods longer than one year might reduce stilbene levels [85]. Most importantly, stilbene production increases in a storage temperature of 15 to 200 °C or after the mechanical process of fresh-pruned canes [86]. Overall, Pinot Noir and Gewurztraminer cane extracts are the richest in stilbenes, containing up to 5800 mg/kg dry weight [82]. The optimization of extraction procedures and parameters, predominantly temperature, is essential; thus, by using subcritical-water extraction, Dorosh et al., 2022, obtained extracts with higher TPC 181 ± 12 mg GAE/g dw from one of the Portuguese vine varieties and noted that the extract with higher flavonoid content had higher TPC and antioxidant activity [87]. Moreover, higher amounts of a non-flavonoid compound, gallic acid (up to 1500 mg of GA/100 g of dw), with known antioxidant activity, were detected [87].

Similarly, grape leaves are another agricultural waste in viticulture and are currently used as soil fertilizers. They contain exciting and abundant antioxidant polyphenols. Studies have shown that the number of polyphenols in red grapevine leaves is similar to that in red grape seeds. The leaf extracts reveal antioxidant, antimicrobial, and antitumor activities [88]. The chemical composition of grape leaves significantly fluctuates according to the development stage, but variety, harvesting period, environmental factors, and soil properties also change the phytochemical profile of the leaves [89]. Although this viticulture waste is less valorized, studies have constantly shown the high antioxidant activity of leaf extracts, which could be due to the synergistic action of various bioactive compounds from different classes (polyphenols, tocopherols, carotenoids, and phytosterols) [88,89]. In recent work on grapevine leaves from 11 different types of vine varieties grown under the same agronomical conditions and in the same soil, Banjanin et al., 2021, revealed that flavonoids, total phenols, and carotenoids are the main bioactive compounds [90]. Another study investigated the phenolic composition of different Pakistani vine varieties from methanolic leaf extracts. The results proved that the variation in soil composition significantly influences the antioxidant potential and TPC of extracts that reached the Shogran-1 variety: 197.58 ± 0.09 GAE/g [91].

Grape canes, stems, and leaf extracts are unexploited sources of natural polyphenols. However, the literature data confirm the presence of over 183 phenolic compounds, including 78 stilbenes, 15 hydroxycinnamic acids, 14 anthocyanins, and other biologically active molecules [92]. Many of these compounds have been shown to modulate human GM in vitro experiments; however, to the best of our knowledge, no published studies exist on the GM modulatory effect of vine by-products. Thus, it has been shown, in vitro, with human probes, that important polyphenolic compounds from grape canes, stilbene resveratrol, and non-flavonoid gallic acid can modulate gut microbiota metabolite production [93,94]. Resveratrol decreases serum TMAO levels via gut microbiota remodeling in mice, reducing bacteria that produce metabolite trimethyl-amine (TMA) and increasing beneficial bacteria such as *Lactobacillus* spp. and *Bifidobacterium* spp. [93]. Moreover, in vitro experiments by Iglesias-Carres et al., 2021, showed the strong TMA-inhibitory potential of gallic acid and chlorogenic acid, indicating a mechanism based on TMA-lyase activity or expression [94]. The GM transforms dietary nutrients such as choline and L-carnitine into TMA, which bacteria oxidize into TMAO through TMA-lyase enzymes. High plasma TMAO levels are associated with atherosclerosis risk. Therefore, this molecule is an important target for decreasing the risk of developing cardiovascular diseases. Various other studies have explored grapevine stem extract’s antimicrobial effect on pathogens such as *Escherichia coli*, *Listeria monocytogenes*, *Staphylococcus aureus*, and *Salmonella* spp. [95]. Similarly, grape leaf ethanolic extract inhibits pathogenic biofilm formation. This activity is related to polyphenolic compounds that suppress the activity of the quorum-sensing system used for cell-to-cell communication [88]. An in vivo study on obese mice fed daily with leaf extract (400 mg/kg) suggested that the bioactive compounds from the extracts prevent obesity by suppressing neuropeptide-Y, which reduces food intake through bile acid mediation. Still, GM alterations were not analyzed [96]. Moreover, grape stem, shoot, and leaf extracts have proven cardioprotective and neuroprotective effects and anticancer, anti-aging, anti-inflammatory, and antioxidant activities [97,98,99,100].

Meaningful observations from in vitro experiments—which indicate the GM modulatory effects of the main polyphenolic compounds from viticulture waste—should be strengthened by in vivo studies of animals and humans. Thus, it is possible to explore their therapeutic potential as health-promoting products and to increase their value, in contrast with the current approach, which uses this waste mainly as fertilizer. Moreover, further research is needed to make vine by-products desirable raw materials and to scale up the extraction process.

## 4. From Winery Waste to Health Products, a New Approach for the Nutraceutical Industry and a New Revenue Stream for Wineries

The food and beverage industry is one of the leading industries that generates different types of waste. Global interest in new valorization processes has increased significantly to protect resources and the environment and for commercial benefit. Various studies on sustainability and environmental impact in the wine industry have shown that the appropriate management and valorization of winery waste is crucial and cheaper than the costs of not recycling [2,22]. This is not a new concept but is an exciting opportunity to optimize winery performance and obtain added-value products by recycling waste streams with valuable ingredients. Since time immemorial, grapevine roots, seeds, and leaves have been used as food preservatives; as flavoring agents with attractive colors; or in traditional medicines. Currently, there are companies (e.g., the Randi Group—Italy; Alvinesa Natural Ingredients—Spain) that transform grape pomace into valuable natural ingredients that promote healthy living (e.g., polyphenols) and other products, such as dyes, anthocyanins, tartaric acid, grape seed oil, and alcohols. High-quality, no-, or low-pesticide GP is the raw material washed in hot water to obtain alcoholic liquid [55]. Next, the alcohol is separated from the tartaric component, and both are used in the food industry; tartaric acid is used as an acidulant or as “cream of tartar” in baking powders, while raw alcohol is used for industrial purposes, and after successive distillation and purification phases, food-grade ethanol is produced [55]. From the exhausted GP, seeds are separated, dried, and pressed to extract crude oil. At the same time, the waste material is used as fuel (GS pellets) or to produce GS flour to be exploited as a dietary supplement or food-enriching ingredient [10,11].

**Figure 1:**
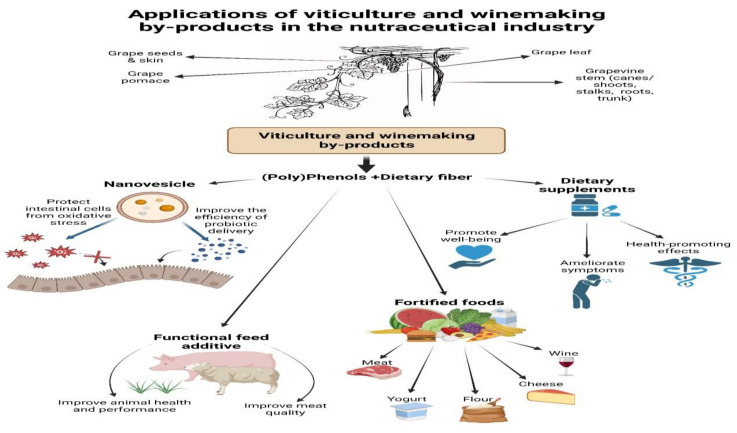
Applications of viticulture and winemaking by-products in the nutraceutical industry.

There is a growing demand for natural compounds that can be used to develop innovative products for the nutraceutical and fortified food industries. Winery by-products might be integrated into this demand due to their phytochemical profiles (e.g., abundant polyphenolic content and fiber), biological properties (e.g., antioxidant properties, anti-inflammatory properties, prebiotic properties for the GM, antimicrobial properties for potential pathogens), and accessibility, thus enhancing wineries’ sustainable development and profitability. Therefore, in the last few years, new applications have been developed (Figure 1). GP is the main agro-waste used by industry. At the same time, polyphenols (4.8–5.4% from GP dry matter) and dietary fiber (29–58% from GP dry weight) are the main components responsible for its health-promoting properties [11]. Conversely, the grapevine stem, which accounts for 25% of total winery waste, is less characterized and exploited from all by-products generated [87].

GP extract, powder, and soaking products in a GP solution are used for yogurt preparation, meat fortification, and cheese production [101,102]. Similarly, food and water can be enriched with GS extract, which increases the antioxidant activity of the product. Grape seed flour is used to bake foodstuffs labeled “low-calorie” [102]. Moreover, considering the restrictions imposed by European Commission legislative regulations, grape flour is safe for consumption [103]. The application of winery by-products to produce fortified foods is in its early stages. Still, considering their health benefits and high nutritional value, there is an opportunity for the development of new foods that boost health. At the same time, GP and GS are suitable ingredients for nutraceutical supplements with antioxidant properties, generally recognized as having no side effects and no interactions with other medicines. These supplements have been used for weight management, to support cardiovascular health, to lower glycemia, for skin protection, etc. A novel nutraceutical formulation based on GP polyphenols, especially catechin and procyanidins (Taurisolo), has significantly reduced TMAO levels and had beneficial vascular effects in different clinical trials [53,104]. Therefore, it has been proposed as an effective nutraceutical for treating high-cardiovascular-risk subjects. Authors have speculated that the observed reduction in TMAO could result in polyphenol-induced microbiota modulation, but further studies are required [63]. To improve our understanding of the capacity of polyphenol-rich winery residue to be used as a GM modulator, large-scale trials are required, as well as research that explores the correlations between complex polyphenol contents and their health-promoting effects. A new, engaging application has been proposed: grape-extract-loaded fiber-enriched vesicles or pomace seed extracts from the Cannonau red grape cultivar loaded into nanovesicles that protect intestinal cells from oxidative stress [105,106,107]. Moreover, polyphenol vesicles obtained from grapes or waste improve the efficiency of probiotic delivery.

Incorporating residual materials from wineries into nutraceuticals is a significant transition toward sustainable health remedies, highlighting the practical benefits, the economic perspectives for comprehending market feasibility, and consumer enlightenment for the promotion of acceptance. This approach emphasizes the possibility of minimizing waste and generating income while also dealing with the need to understand and comply with regulations and establish oneself on the market. Ultimately, it aims to foster a healthier and more sustainable future. Therefore, integrating winery by-products into nutraceuticals is in line with the concept of the circular economy, providing a sustainable approach to the use of resources. This strategy optimizes the use of vineyard byproducts by transforming them into premium health commodities, thereby minimizing waste and fostering environmental sustainability. This highlights the possibility of generating economic prospects in the viticulture and health industries while also meeting customer needs for environmentally friendly and health-enhancing goods. This collaboration improves the effectiveness of resource use, promotes economic development, and cultivates a culture that is more environmentally friendly and mindful of well-being.

## 5. Conclusions

There are concluding reports that grapevine waste polyphenol-rich extracts and diet supplementation have caused an ecological shift in the microbiome related to the health-promoting effects reported. However, the effect of grapevine-waste-derived products on gut microbiota is a challenge to study, as it is complex, and the current state of knowledge does not provide a mechanism for studies. Knowledge of this subject is progressing with the experimental procedure and analysis techniques. However, further research is needed to explore the complex relationship between GM and grapevine waste polyphenol-rich extracts.

Overall, the diverse array of bioactive phenolic compounds from viticulture and winery waste have vast potential to be exploited by the nutraceutical industry. Further microbiota research should focus on grapevine stems, which are the least valorized subproduct of the wine industry despite being produced in massive amounts. This is a valuable source of resveratrol, which influences the composition of GM, fostering microbial diversity and inducing a balance that supports overall health. Also, research should focus on extracting and concentrating procedures for bioactive components that provide targeted interventions for improving gut health and inducing health-promoting effects.

New therapeutic applications of grapevine-waste-derived products will contribute to sustainable waste management and the development of functional ingredients that positively impact human health, and they could be a new revenue stream for wine producers.

## Figures and Tables

**Table 1:** In vitro and in vivo studies on the effects of grape pomace extracts and supplemented diets on the gut microbiota composition and metabolite profile.

Source/Polyphenol/Quantity	Model/Conditions/ Control	Impact on Microbiota Composition and Metabolites Profile/Health Benefits	Ref.
Humans			
GP extract rich in gallic and ellagic acids, catechins, and flavonols from red grapes	In vitro human feces fermentation	Promotes the growth of gut microbiota, especially the Enterococcus group; Sixteen phenolic bacterial metabolites produced.	[36]
GP extract from red grapes (Eminol^®^) in 2 experiments: acute feeding of 700 mg and continuous feeding of 700 mg/day for 14 days	In vitro colonic microbiota simulation (dynamic)	After continuous treatment: Increases *Lactobacillus* and *Bacteroides* group; Increases SCFAs (acetic, propionic, and butyric acids); Main bioaccessible phenolic metabolites: different benzoic, phenylacetic, and phenylpropionic acids.	[14]
Red GP extract (Eminol^®^) (1.4 g/day)-supplemented diet for 21 days	Human trial with healthy women (*n* = 10 subjects)	No significant changes in fecal bacterial population or urine phenolic metabolites; Changes in SCFAs and medium-chain fatty acid profiles; Inter-individual variability observed; Lowers the glucose level and miRNA modulation of glucose metabolism.	[37]
GP seasoning (2 g/day) for 6 weeks	Human trial with high-risk cardiovascular disease (*n* = 17 subjects) and normal subjects (*n* = 12 subjects)	Bacterial composition modulation (16S rRNA sequencing); Decreases *Peptoniphilus*, *Clostridiaceae* I, *Ezakiella*, *Streptococcus*, *Lachospiraceae* ND3007, *Paraprevotella*, *Senegalimassilia*, *Streptococcaceae*, and *Eggerthellaceae*, *Corinebacteriales*; Increases protocatechuic acid and decrease propionic acid; Blood pressure and glycemia decreased; GP extract could be a promising additive in food.	[11]
Red GP from Tempranillo grape variety, freeze-dried and milled (8 g/day) for 6 weeks	Human trial with overweight subjects at cardiometabolic risk (*n* = 49 subjects); cross-over design study	No significant modifications in the microbiota profile or SCFA; Reduction in insulin levels in half of the subjects (responders), which was reported to be not induced by changes in the gut microbiota; Decreases Lactobacilliales and increases *Bacteroides* in non-responder subjects.	[38]
Red GP from Tempranillo grape variety, freeze-dried and milled (8 g/day) for 6 weeks	Human trial with overweight subjects at cardiometabolic risk (*n* = 49 subjects) divided into responders (*n* = 23) and non-responders (*n* = 26) based on the variation rate in fasting insulin after GP supplementation	Lower levels of *Prevotella* and Firmicutes, while miR-222 levels increased in responder subjects; Fecal microbiota and miRNA expression may be related to inter-individual variability in clinical trials with polyphenols.	[39]
Pigs and piglets			
5% GP proanthocyanidin-rich extract (industrial product) for 14 days	Danish Landrace–Yorkshire pigs, 7–8 weeks old, infected with *Ascaris suum* (*n* = 6 animals/group) Groups: 2 groups fed with basal diet, barley and soybean meal (control), and 2 groups with basal diet + GP 5%	Changes the composition of the prokaryotic gut microbiota; Decreases concentrations of isobutyric and isovaleric acids (branched-chain SCFAs) in the colon; Improves the immune response but does not decrease the number of parasite larvae.	[40]
6% GP powder (dried) in basal diet for 75 days	Guanzhong Black Pig × Landrace pigs (*n* = 12 animals/group) Groups: basal diet (control) and group fed with 6% wheat bran and 6% dried GP powder	No differences between the control and the treatment group at the phylum level; Decreases the relative abundance of *Treponema* and *Streptococcus,* but no changes for other bacterial genera (*Prevotella*).	[41]
4% red GP-supplemented diet for 30 days	Landrace–large white Duroc piglets (*n* = 12 animals/group) Groups: basal diet (control); diet + GP silage	Enhances the growth of facultative probiotic bacteria and lactic acid bacteria; Inhibits *Enterobacteriaceae* and *Campylobacter jejuni*; Increases antioxidant activity, beneficial impact on piglet welfare, and improves productivity and meat quality.	[42]
5% red GP-supplemented diet for 4 weeks	Songliao black pigs, 28-day old (*n* = 6 animals/group) Groups: basal diet (control), diet + GP	Increases *Lactobacillus delbrueckii*, *Olsenella umbonata*, and *Selenomonas bovis* Improves the disease resistance potential of piglets	[43]
Lambs			
45% GP-supplemented diet for 55 days	Chios lambs, 15 days old (*n* = 12 animals/group) Groups: basal diet (control); diet + GP	Enhances the growth of facultative probiotic bacteria; Inhibits pathogen populations (*Enterobacteriaceae* and *E. coli*); Increases antioxidant mechanisms in blood and tissues.	[18]
8% red GP-supplemented diet for 46 days	Tan lambs, 3 months old (*n* = 10/group) Groups: basal diet (control); diet + GP	Increases the abundance of *Prevotella* 1, *Prevotella* 7, *Ruminococcus* 2, *Sharpea*, and microbial propionate production; Decreases the acetate-producing *Ruminococcaceae* and methane-producing *Methanobrevibacter*; Improves lamb performance.	[44]
Calves			
Red GP (10% dry matter)-supplemented diet for 75 days	Holstein–Friesian calves, 7 months old (*n* = 5/group) Groups: basal diet (control), diet + GP; diet + copper supplementation	Increases the diversity of the lumen microbiota and alters gene functions; Abundant taxa are uncultured Bacteroidales, genus *Sarcina*, and taxa related to degradation of GP constituents (*Eubacterium* sp., *Ruminiclostridium* sp.).	[45]
Chickens			
GP concentrate (60 g/kg) for 21 days	Cobb broiler chickens (*n* = 25 animals/group) Groups: antibiotic-free (control), antibiotic-included, and GP included	Increases the biodiversity; Increases *Enterococcus* and decreases *Clostridium* (ileum); Increases *E. coli, Lactobacillus, Enterococcus*, and *Clostridium* (cecum).	[15]
2.5% GP-supplemented diet for 42 days	Cobb-500 broiler chickens, 1 day old (*n* = 192 animals/group) Groups: antibiotic-free (control), antibiotic included, and GP included	Increases *Bacteroidetes*, *Bacteroides*, and *Lactobacillus* (cecum); Decreases Firmicutes; SCFA (cecum) does not change; Increases feed intake, but feed conversion ratio does not change; No adverse effects on animal growth or meat quality.	[46]
Rodents			
GP phenolic extract mix of Cabernet Sauvignon, Marselan, and Syrah varieties (2.5–20 mg/kg body weight/day) for 6–14 months	Wistar rats, 2 months old (*n* = 6 animals/group) Groups: 0.1% DMSO (control); GP included	Increases *Bifidobacterium* (only treatment with 2.5 and 5 mg/kg body weight/day); Decreases *Lactobacillus*; No changes in *Bacteroides*, *Enterococcus*, or *Clostridium leptum* subgroup (Clostridium cluster IV) but abolished the increase in *Clostridium* Cluster I; Long-term treatment modulates rat gut microbiota into a healthier phenotype within age.	[16]
GP red extract powder (2.5–20 mg/kg body weight/day) in drinking water for 14 months	Wistar rats (*n* = 6 animals/group) Groups: 0.1% DMSO (control); GP-included	Long-term intake of GP seems to be related to specific metabolites produced; Sixteen polyphenol-derived metabolites detected; Increases *Bifidobacterium* correlated with the presence of two metabolites (3-hydroxyphenylacetic acid and 2-(4-hydroxyphenyl) propionic acid); *Clostridium perfringens* (Cluster I) inhibition with Daidzedin.	[17]
Red GP extract (8.2 g/kg body weight)	C57BL/6J mice, 9 weeks old, fed a high-fat diet for 8 weeks (*n* = 14 animals/group) Groups: control, high-fat diet, and high-fat + GP diet	Decreases in *Desulfovibrio* and *Lactococcus*; Increases *Allobaculum* and *Roseburia*; Improvement of gut barrier function; Increases antimicrobial peptide; Reduction in fat mass gain and adipose tissue inflammation; Improves glucose tolerance and lowers insulin resistance index.	[47]
GP extract from Kyoho blake grape (200 mg/kg body weight) after a 3-week antibiotic treatment	C57BL/6J mice, 9 weeks old, fed a high-fat diet and antibiotic treatment (*n* = 10 animals/group) Groups: saline solution (control)	Contributes to the recovery of gut microbiota after antibiotic treatment, increasing its composition and improving its complexity on the genus level; Increases relative abundance of *Akkermansia*, and *Alloprevotella* Does not restore *Bifidobacterium*, *Lactococcus*, or *Lactobacillus*.	[13]

GP—grape pomace.

**Table 2:** In vitro and in vivo studies on the effects of grape seed extracts and supplemented diets on gut microbiota composition and metabolite profile.

Source/Polyphenol/Quantity	Model/Conditions/ Control	Impact on Microbiota Composition and Metabolites Profile/Health Benefits	Ref.
Humans			
GS extract polyphenols obtained by pressurized liquid extraction and purification with macroporous resin	In vitro human feces fermentation for 36 h	Increases *Bifidobacterium* spp. and *Lactobacillus*–*Enterococcus* group changes in SCFA profiles; Inhibits *Clostridium histolyticum* group and the *Bacteroides*–*Prevotella* group; No significant effect on the population of total bacteria.	[59]
Two purified fractions from GE extract: GSE-M (70% monomers and 28% procyanidins) and GSE-O (21% monomers and 78% procyanidins)	In vitro human feces fermentation for 48 h	During the first 10 h of fermentation, GSE-M fractions significantly promote growth of *Lactobacillus*–*Enterococcus*, and GSE-O decreases in the *Clostridium histolyticum* group; Microbial precursor flavan-3-ol could affect the microbiota composition and its catabolic activity.	[60]
GS and cranberry extracts	In vitro colonic microbiota simulation	Antimicrobial effect of GS polyphenols on *Bacteroides*, *Prevotella*, and *Blautia coccoides*–*Eubacterium rectale*	[61]
Proanthocyanidin-rich GS extract	Human fecal flora and odor (healthy adults)	Increases *Bifidobacterium*	[62]
Pig and piglets			
Proanthocyanidin-rich GS extract—MegaNatural^®^ Gold (1% (*w*/*w*) for 6 days	Pigs, crossbred, female (*n* = 6 animals) Groups: basal diet (control); diet + GSE	Increases *Lachnospiraceae*, *Clostridales*, *Lactobacillus* and *Ruminococcacceae * Phenolic metabolites 4-hydroxyphenylvaleric acid and 3-hydroxybenzoic acid major increased	[63]
GS and skin polyphenol-rich extract (100 ppm) and a low dose of functional amino acids (0.1%) during the first 2 weeks	Landrace x large white x Duroc piglets, 25 days old (*n* = 6 animals/group) Groups: basal diet (control), diet + GSE, and diet + amino acids mix	Reduces microbiota diversity; Highly increased Lactobacillaceae in the jejunum; Reduces the abundance of Proteobacteria in the caecum; Increases the production of SCFAs (butyrate and propionate) and of metabolites derived from amino acids (branched-chain fatty acids, valerate, and putrescine) and polyphenols (3-phenylpropionate); Improves growth and feed efficiency.	[64]
GS procyanidin extract (50, 100, and 150 mg/kg body weight) for 28 days	Duroc × Landrace × Yorkshine piglets, 21 days old (*n* = 24 animals/group) Groups: basal diet (control); diet + GSE (50, 100, and 150 mg/kg)	Increases diversity indices (Ace, Chao1) of cecal, colonic, and rectal microflora (100 mg/kg body weight dose); Increases *Prevotellaceae* NK3B31 and *Prevotella* I Decreases *Proteobacteria* and *Anaerovibrio* Increases SCFAs: propionic acid and, especially, acetic acid and butyric in the cecum and colon (100 mg/kg body weight dose); Increases antioxidant capacity.	[65]
Chickens and hens			
GS extract (1–2%)-supplemented diet for 40 weeks	Cobb 500-broiled hens, 40 weeks old (*n* = 12/group) Groups: basal diet (control), diet + GSE 1%, and diet + GSE 2%	Increases relative abundance of *Bifidobacteriaceae*, *Lactobacilliaceae*, and *Lachnospiraceae*; Improves metabolic and laying parameters (decreases fat tissue; improves fertility; and makes eggs more resistant).	[66]
GS extract (7.2 g/kg) for 21 days	Cobb broiler chickens (*n* = 25 animals/group), Groups: antibiotic-free (control), antibiotic-included, and GP included	Increases biodiversity; Increases *Enterococcus* and *Lactobacillus*, and decreases *Clostridium* (ileum); Increases *E. coli*, *Lactobacillus*, *Enterococcus*, and *Clostridium* (cecum); Decreases weight gain.	[15]
GS extract (10, 20, and 40 g/kg of diet), daily for 42 days	Cobb-500 broiler chicks, 1 day old (*n* = 300/group) Groups: basal diet (control); diet + GSE (10, 20, and 40 g/kg)	Decreases detrimental bacteria in the ileum, *E. coli*, and Streptococcus; Increases *Lactobacillus*; Improves performance; increases body weight.	[67]
Fermented GS meal (2–6%) for 56 days	Broiler chicks, 14 days old (*n* = 80/group) Groups: basal diet (control); diet + GSE (2%, 4%, and 6%)	Increases biodiversity indices (Shannon and Simpson); Reduces relative abundance of Bacteroidetes (cecum); Increases relative abundance of Firmicutes (cecum); Increases butyric acid content after a 4% fermented GS-meal-supplemented diet; Strong correlation between broiler growth performance, abdominal fat percentage, SCFAs, and gut microbiota; Improves broiler growth performance and reduces fat deposition.	[68]
Rodents			
Proanthocyanidin-rich GS extract (300 mg/kg body weight) for 7 weeks	Mice fed a high-fat diet (HFD) (*n* = 10–12) Groups: HFD + PBS (control); HFD + GSE	Modulate *Clostridium* XIVa, *Roseburia*, and *Prevotella*; Decreases plasma inflammatory factors, reduces epidydimal fat mass, and improves insulin sensitivity; Link between gut microbiota alterations and metabolic benefits of GS extract supplementation.	[69]
Proanthocyanidin-rich GS extract for 8 weeks	D-galactose (500 mg/kg)-induced aging mice Groups: basal diet (control); diet + GSE	Increases *Lachnospiraceae* NK4A136, *Lactobacillus*, *Bifidobacterium*, and *Akkermansia*; Decreases *Helicobacter* and *Alistipes*; Improves antioxidant capacity and inflammation levels in the liver and brain; High-dose extract has greater potential to delay aging process via the gut microbiota–liver axis and gut–microbiota–brain axis.	[70]
Proanthocyanidin-rich GS extract (25 mg/kg body weight/day) for 9 weeks	Cafeteria-diet-induced obese Fischer 344 rats exposed to photoperiod conditions (6 h, 12 h, and 18 h) (*n* = 10 animals/group)	GS extract decreases body weight gain and fat deposits, and GM strongly altered under 18 h of light; GS extract functionality is modulated by the GM in a photoperiod-dependent manner.	[71]
Proanthocyanidin-rich GS extract (200 mg/kg body weight/day) for 8 weeks	C57BL/6J mice fed with high-fat diet (*n* = 10 animals/group) Groups: HFD (control); HFD + GSE	Improves diversity; Normalized Firmicutes/Bacteroidetes ratio; Reverses the relative abundance of *Weissella*, *Faecalibaculum*, *Bacteroides*, *Akkermansia*, and *Ruminococcus* 1 induced by high-fat diet; Increases acetic acid, propionic acid; and butyric acid in the colon; Reduces the final body weight and associated metabolic complications.	[72]
Proanthocyanidin-rich GS extract (250 mg/kg body weight/day) for 20 days	C57BL/6J mice, 7–8 weeks old, injected with LPS to induce intestinal inflammation Groups: basal diet (control), diet + GSE	Alters gut microbiota; Increases abundance of hydroxysteroid dehydrogenase-producing microbes; Extract shows an intestinal protection role in the inflammation induced by LPS; effect mediated by regulating the GM.	[73]
GS extract after a 3-week antibiotic treatment	C57BL/6J mice, 9 weeks old, fed a high-fat diet and antibiotic treatment (*n* = 10 animals/group) Groups: saline solution (control)	Contributes to the recovery of gut microbiota after antibiotic treatment; Increases relative abundance of *Akkermansia*, *Alloprevotella*, and *Prevotella*; Does not restore beneficial bacteria (*Bifidobacterium*, *Lactococcus*, and *Lactobacillus*).	[13]
GS and skin extract (GSSE) (4 g/kg body weight for 3 months) GSSE + orlistat (medication used to treat obesity)—GSOR treatment	Rats (obese), 12 weeks old, fed with high-fat diet (*n* = 24 animals/group) Groups: standard diet and HFD (controls); GSSE, GSOR, and GSSE + GSOR groups	Biodiversity restores with GSSE and GSOR treatments; Increases beneficial microbes (*Methanobrevibacter*, *Ruminococcus* 2, *Lachnospiraceae* NK4A136); Decreases pathobionts, especially *Streptococcus alactolyticus*/*gallolyticus, Enterobacteriaceae* (*Escherichia* genus), *Allobaculum*, *Turicibacter*, and *Tyzzerella* 3; Partially restores dysbiosis produced by obesity; Decreases body weight and fat accumulation and improves serum lipid parameters, especially the GSOR treatment.	[74]
Proanthocyanidin-rich GS extract—GSPE (0.5 g/kg body weight/day) for 8 days	Wistar rats (female) (*n* = 9 animals/group) Groups: control, diet + GSPE, and diet + gallic acid	Increases Bacteroidetes; Reduces Firmicutes and cecal butyrate; Correlation between microbiota modulation and plasma triacylglycerol, adiposity, and enterohormone secretion.	[75]
Canine			
Proanthocyanidin-rich GS extract (30 mg/kg body weight) for 28 days	Canine adult Labrador retrievers with inflammatory bowel disease (IBD) (*n* = 12 healthy subjects, *n* = 24 subjects with IBD)	Increases *Ruminococcaceae*, *Faecalibacterium*, *Ruminococcus torques* group, and *Lachnospiraceae* NK4A136 group; Improves inflammatory index and reduces intestinal permeability; Extract’s effect on canine inflammation is related to alteration of GM and microbiota-derived bile acids.	[76]
Animal in vitro studies			
Proanthocyanidin–GS extract (15–120 mg/g of substrate)	In vitro rumen	Decreases the ratio of methanogens to total bacteria, especially for *Methanobrevibacter*; Extract decreases methane emissions.	[77]

GS—grape seed.

## Data Availability

Not applicable.
